# Reproductive outcomes among female health care workers

**DOI:** 10.1186/s12905-024-02890-x

**Published:** 2024-01-16

**Authors:** Nazanin Izadi, Omid Aminian, Kiana Ghafourian, AmirHossein Aghdaee, Shadi Samadanian

**Affiliations:** 1 Center for research on occupational diseases, Tehran University of medical sciences, Tehran, Iran; 2 School of medicine, Shahid Beheshti University of Medical Sciences, Tehran, Iran; 3 Infectious Diseases and Tropical Medicine Research Center, Shahid Beheshti University of Medical Sciences, Tehran, Iran

**Keywords:** Breastfeeding, Health care workers, Occupational exposure, Adverse pregnancy outcomes, Reproductive health, Shift work

## Abstract

**Objective:**

Occupational exposures may be associated with reproductive health and pregnancy outcomes. This study investigated the association between occupational exposures and reproductive health, pregnancy outcomes, and the lactation period among hospital staff.

**Materials and methods:**

Seven hundred thirty-three female healthcare workers from hospitals affiliated with the Tehran University of Medical Sciences were invited to participate in this cross-sectional study. The measurement method for fertility consequences was self-report. Demographic characteristics, occupational data, medical history, and reproductive history were collected via data collection form. Finally, reproductive outcomes were evaluated in different occupational hazard categories.

**Result:**

Chemical exposures (solvents) were a risk factor for stillbirth. Prolonged working hours were associated with spontaneous abortion and breastfeeding periods. Shift workers did not have a higher frequency of reproductive and pregnancy outcomes, but the breastfeeding period was significantly decreased in shift workers. Psychiatric disorders were associated with preterm labour, low birth weight, and stillbirth in sequence with nervousness, depression, and mood disturbance. Furthermore, depression affects the breastfeeding period. Moreover, we found a link between job titles and infertility. In addition, socioeconomic status was related to stillbirth and infertility.

**Conclusion:**

The study revealed that chemical and ergonomic exposures have associations with some reproductive outcomes. We also conclude that shift work could adversely affect the breastfeeding period. So, implementing some organizational strategies to control adverse health effects of occupational hazards and modifying shift work and working hours for nursing mothers is recommended.

## Introduction

 There has been an increase in women’s employment over recent years, so a large percentage of the labor force in industrialized countries are women [1, 2]. According to the BLS (Bureau of Labor Statistics) data book report, women comprised 57.4% of the work force in the United States, with a majority employed in the following job categories: office and administrative support, education/library, health care provider, and personal care and service. There are various hazards in different occupational groups. For instance, the health industry encounters occupational hazards, including chemicals (anesthetic gas, solvents, antineoplastic drugs ), physical (ionizing and non-ionizing radiation), ergonomic (long working hours, long-standing, lifting and carrying heavy loads or patients) psychiatric (shift work, stress, violence) and biologic hazards [3]. Physical exertion at work has been a cause of concern especially on women during pregnancy [4, 5] .The American College of Obstetricians and Gynecologists published guidelines on exertion levels during pregnancy, indicating that heavy workload, prolonged standing, or repetitive bending are recommended to be discontinued early during the second trimester. The CDC reported that the most consistent adverse effect of physical exertion seems to be on preterm delivery and possibly LBW and SAB, with less consistent results for fecundability and menstrual disorders [6]. . However, there is conflicting evidence about whether work schedules, including shifts, can affect fertility outcomes [7]. Adverse pregnancy outcomes range from infertility to congenital disabilities in the infant, which include spontaneous abortion, stillbirth, preterm labor, low birth weight, and IUGR [8, 9]. Infertility is a reproductive outcome estimated that about 15.5% of women in the United States are infertile, and a wide range of behavior, mood, and exposures are associated with this decline in fertility rate. Infertility is often defined as being unable to get pregnant after one year of unprotected intercourse [10]. Additionally, couples may experience periods of subfertility or delayed conception. Of all live-born infants, 7–9% have low birth weight (LBW, and approximately 11% are born prematurely.

Today, many countries are in a state of population imbalance which is caused by negative population growth and aging. Considering the fact that employment in women is potentially one of the obstacles to fertility and about 50% of women become pregnant during their employment, especially in active years of work, [11] and the emotional, medical and social burden, it is important to identify and control external factors such as hazardous work environment.

As mentioned, working in health care industry is associated with various occupational hazards. Due to the importance of this issue and the few articles on reproductive outcomes in healthcare workers especially in Iran, we decided to investigate the relationship between reproductive health and pregnancy outcomes with the physical, psychological, chemical and ergonomic hazards encountered by healthcare workers at work. The extent of reproductive outcomes and occupational risk factors obtained in this study could provide a framework for future studies.

## Materials and methods

### The study population

This cross-sectional study was performed on 733 female healthcare workers chosen with a simple randomized method, in hospitals affiliated with the Tehran University of Medical Sciences (Iran) from April 2021 to January 2022.

### Inclusion and exclusion criteria

Married for more than a year female employee with at least three years of working history are included in our study. The exclusion criteria were unwillingness to participate in the study, participants with no desire to have children, and missing data.

The data was gathered anonymously; participation was voluntary and informed consent was obtained from all study participants.

### Definition and measurements

The data-gathering form was presented to the participants based on a self-report with the following items. It included demographic criteria (age, height and weight, marital status and socioeconomic level), educational and occupational characteristics (level of education, job title, work experience by year, working hours/week, working in shift and different occupational exposures including anesthetic gases, anticancer drugs, solvents, ionizing and non-ionizing radiation and ergonomically hazards), different items of reproductive health and outcomes and its known risk factors (first pregnancy’s age, number of pregnancies, number of children, number of abortions, still birth, low birth weight and IUGR and use of contraceptives or not and history of infertility and its treatment, breastfeeding period by months and age of menopause if it occurs), health status including past medical history (history of renal, cardiac, endocrine, immunosuppressive and gynecological diseases/surgery and cancers), drug history and familial history, mental health and psychiatric status (depression, anxiety, nervous ), diet status (fast food as high fat- high carb or safe diet as low fat-low carb and consumption of tea, coffee, Chocolate and Soft drink), habitual history (smoking cigarettes and consumption of alcohol), level of physical activity (based on frequency in the week) and etc. They also reported the level of extended sitting or/and standing and heavy work in their job using the following categories: light (e.g., most time spent sitting, office work), moderate (e.g., lifting/pushing light loads, long periods of walking), and heavy (e.g., lifting, pushing heavy loads, heavy manual labor).

### Statistical methods

The relationship between occupational hazards (chemical, physical, ergonomic, and psychiatric) and reproductive outcomes was determined based on all demographic, socio-economic, and other variables. We used IBM Corp. Released 2019. IBM SPSS Statistics for Windows, Version 26.0. Armonk, NY: IBM Corp to perform the analysis. Quantitative and qualitative data were expressed as mean (SD) and frequency (percent). In Univariate analysis, one-way *ANOVA* and Chi-square were *used*. Additionally, Logistic regression was performed to find the related factors of reproductive outcomes, and linear regression was used for associated factors of the breastfeeding period.

### Ethical consideration

The study was approved by the Ethics Committee of Tehran University of Medical Sciences (ethical code: IR.TUMS.IKHC.REC.1399.249) and follow all principals of Helsinki declaration. The informed consent was obtained from the study participants.

## Results

The study population consists of 789 female healthcare workers. Of these, 56 women (7.09%) were excluded because of incomplete data or discrepancies between answers. The mean (SD) of age and working history were 35.01(7.49) and 10.52(6.38), respectively.

**Fig. 1:**
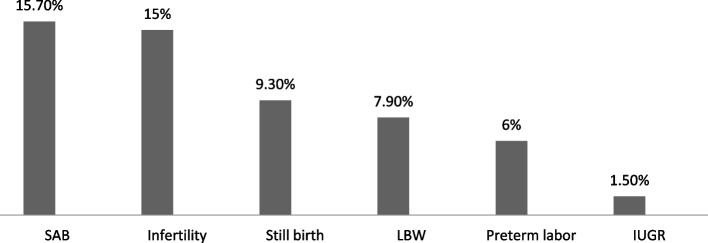
The frequency of reproductive outcomes (percent)

**Fig. 2:**
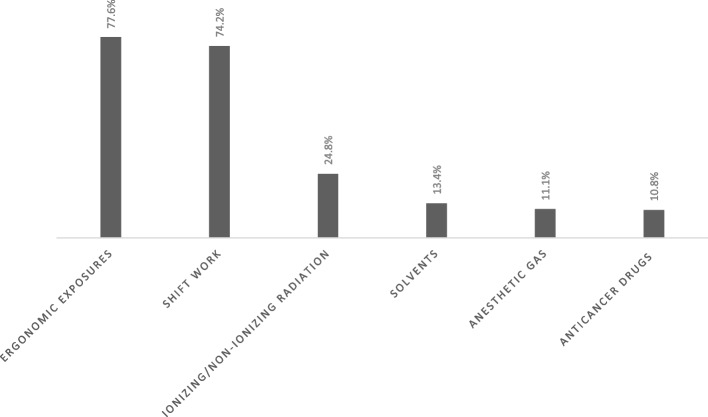
The frequency of different occupational hazards

**Fig. 3:**
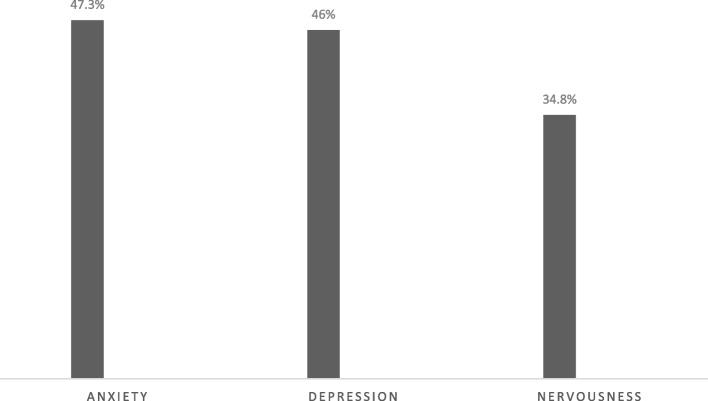
The frequency of mood disturbance

The baseline characteristics of the study population are presented in Table 1. The frequency of spontaneous abortion, infertility, stillbirth, low birth weight, preterm labor, and IUGR was 115 (15.7%), 110(15%), 68 (9.3%), 58 (7.9%), 44 (6.0%), 11 (1.5%) in sequence (Fig. 1). The frequency of occupational hazards and mood disturbance is shown in Figs. 2 and 3, respectively. The association between different reproductive outcomes with occupational hazards, diet and habitual history, mood status and past medical history, and reproductive history is shown in Tables 2, 3, 4 and 5, respectively. Logistic regression analysis indicated the related factors of each reproductive outcome (Table 6). Linear regression analysis showed a significant positive association between decreased lactation periods, shift work, prolonged working hours, and depression (Table 6) (*P*.VALUE < 0,05).

**Table 1:** The association between different reproductive outcomes with demographic and occupational characteristic *: *p* < 0.05

		Infertility			SAB		Stillbirth		preterm labor		IUGR		LBW	
		No		Yes	No	Yes	No	Yes	No	Yes	No	Yes	No	Yes
**Quantitative Variables mean (SD)**														
**Age**		35.19(7.62)		33.98(6.67)	35.01(7.44)	35.03(7.81)	35.08(7.48)	34.31(7.66)	35.08(7.51)	34.00(7.32)	35.19(7.62)	33.98 (6.67)	35.19(7.62)	33.98(6.67)
**BMI**		24.43(3.86)		23.75(3. 78)	24.34(3.87)	24.26(3.78)	24.37(3.85)	23.86(3.85)	24.31(3.80)	24. 56(4.64)	24.43(3.86)	23.75 (3.78)	24.43(3.86)	23.75(3.78)
**Working hr/wk**		43.29(10.07)		44.65(9.42)	43.26(10.18)	44.77(8.75)	43.74(10.03)	41.10^*^(9.25)	43.42(10.02)	44.68(9.34)	43.29(10.07)	44.65 (9.42)	43.29(10.07)	44.65(9.42)
**Working Year**		10.74(6.47)		9.32^*^(5.71)	10.37(6.36)	11.33(6.46)	10.45(6.34)	11.26(6.71)	10.56(6.39)	9.98(6.23)	10.57(6.40)	7.09(3.44)	10.45(6.37)	11.41(6.48)
**Income**		7.26(5.23)		7.21(5. 40)	7.23(5. 30)	7.36(4.98)	6.98(4.40)	9.89^*^(10.07)	7.23(5.33)	7.58(3.69)	7.26(5.23)	7.21(5. 40)	7.26(5.23)	7.21(5.40)
**Qualitative Variables n (%)**														
**Socioeconomic**	low to moderate	485(77.8)	97(88.2)		482(82.8)	100(17.2)	530(91.1)	52(8.9)	545(93.6)	37(6.4)	572(79.2)	10(90.9)	530(78.5)	52(89.7)
	high	138(22.2)	13^*^(11.8)		136(90.1)	15^*^(9.9)	135(89.4)	16(10.6)	144(95.4)	7(4.6)	150(20.8)	1(9.1)	145(21.5)	6^*^(10.3)
**Job title**	Office	68(10.9)	5(4.5)		59(80.8)	14(19.2)	66(90.4)	7(9.6)	73(100.0)	0(0.0)	73(10.1)	0(0.0)	70(10.4)	3(5.2)
	Clinical	555(89.1)	105^*^(95.5)		559(84.7)	101(15.3)	599(90.8)	61(9.2)	616(93.3)	44^*^(6.7)	649(89.9)	11(100.0)	605(89.6)	55(94.8)
**Shift work**	No	166(26.6)	23(20.9)		165(87.3)	24(12.7)	170(89.9)	19(10.1)	184(97.4)	5(2.6)	187(25.9)	2(18.2)	178(26.4)	11(19.0)
	Yes	457(73.4)	87(79.1)		453(83.3)	91(16.7)	495(91.0)	49(9.0)	505(92.8)	39^*^(7.2)	535(74.1)	9(81.8)	497(73.6)	47(81.0)
**Educational degree**	≤Bachelor	481(84.5)	88(15.5)		471(82.8)	98(17.2)	514(90.3)	55(9.7)	538(94.6)	31(5.4)	561(77.7)	8(72.7)	524(77.6)	45(77.6)
	>Bachelor	142(86.6)	22(13.4)		147(89.6)	17^*^(10.4)	151(92.1)	13(7.9)	151(92.1)	13(7.9)	161(22.3)	3(27.3)	151(22.4)	13(22.4)

*: *p* < 0.05

**Table 2:** The association between different reproductive outcomes with occupational hazards

Variables n (%)		Infertility		SAB		Stillbirth		preterm labor		IUGR		LBW	
		No	Yes	No	Yes	No	Yes	No	Yes	No	Yes	No	Yes
**Anesthetic gases**	No	555(89.1)	97(88.2)	552(84.7)	100(15.3)	592(90.8)	60(9.2)	611(93.7)	41(6.3)	641(88.8)	11(100.0)	599(88.7)	53(91.4)
	Yes	68(10.9)	13(11.8)	66(81.5)	15(18.5)	73(90.1)	8(9.9)	78(96.3)	3(3.7)	81(11.2)	0(0.0)	76(11.3)	5(8.6)
**Anti-cancer drugs**	No	556(89.2)	98(89.1)	550(84.1)	104(15.9)	593(90.7)	61(9.3)	616(94.2)	38(5.8)	644(89.2)	10(90.9)	605(89.6)	49(84.5)
	Yes	67(10.8)	12(10.9)	68(86.1)	11(13.9)	72(91.1)	7(8.9)	73(92.4)	6(7.6)	78(10.8)	1(9.1)	70(10.4)	9(15.5)
**Solvent**	No	538(86.4)	97(88.2)	541(85.2)	94(14.8)	583(91.8)	52(8.2)	594(93.5)	41(6.5)	625(86.6)	10(90.9)	583(86.4)	52(89.7)
	Yes	85(13.6)	13(11.8)	77(78.6)	21(21.4)	82(83.7)	16^*^(16.3)	95(96.9)	3(3.1)	97(13.4)	1(9.1)	92(13.6)	6(10.3)
**Gamm & X Ray radiation**	No	467(75.0)	84(76.4)	469(75.9)	82(71.3)	503(91.3)	48(8.7)	523(94.9)	28(5.1)	544(75.3)	7(63.6)	507(75.1)	44(75.9)
	Yes	15(25.0)	26(23.6)	149(24.1)	33(28.7)	162(89.0)	20(11.0)	166(91.2)	16(8.8)	178(24.7)	4(36.4)	168(24.9)	14(24.1)
**Standing sitting**	No	80(12.8)	14(12.7)	81(13.1)	13(11.3)	86(12.9)	8(11.8)	88(12.8)	6(13.6)	93(12.9)	1(9.1)	89(13.2)	5(8.6)
	Long Standing	220(35.3)	33(30.0)	213(34.5)	40(34.8)	229(34.4)	24(35.3)	239(34.7)	14(31.8)	250(34.6)	3(27.3)	233(34.5)	20(34.5)
	Long sitting	192(30.8)	43(39.1)	191(30.9)	44(38.3)	210(31.6)	25(36.8)	216(31.3)	19(43.2)	232(32.1)	3(27.3)	215(31.9)	20(34.5)
	Both Standing & sitting	131(21.0)	20(18.2)	133(21.5)	18(15.7)	140(21.1)	11(16.2)	146(21.2)	5(11.4)	147(20.4)	4(36.4)	138(20.4)	13(22.4)

*: *p* < 0.05

**Table 3:** The association between different reproductive outcomes with diet and habitual history

Variables n (%)		Infertility		SAB		Stillbirth		preterm labor		IUGR		LBW	
		No	Yes	No	Yes	No	Yes	No	Yes	No	Yes	No	Yes
**Safe diet**	No	185(29.7)	32(29.1)	185(85.3)	32(14.7)	203(93.5)	14(6.5)	202(93.1)	15(6.9)	212(29.4)	5(45.5)	195(28.9)	22(37.9)
	Yes	438(70.3)	78(70.9)	433(83.9)	83(16.1)	462(89.5)	54(10.5)	487(94.4)	29(5.6)	510(70.6)	6(54.5)	480(71.1)	36(62.1)
**Fast Food**	No	544(86.1)	88(13.9)	532(84.2)	100(15.8)	579(91.6)	53(8.4)	590(93.4)	42(6.6)	623(86.3)	9(81.8)	579(85.8)	53(91.4)
	Yes	79(78.2)	22^*^(21.8)	86(85.1)	15(14.9)	86(85.1)	15^*^(14.9)	99(98.0)	2(2.0)	99(13.7)	2(18.2)	96(14.2)	5(8.6)
**Multivit amin consumption**	No	317(85.9)	52(14.1)	309(83.7)	60(16.3)	336(91.1)	33(8.9)	348(94.3)	21(5.7)	363(50.3)	6(54.5)	335(49.6)	34(58.6)
	Yes	306(84.1)	58(15.9)	309(84.9)	55(15.1)	329(90.4)	35(9.6)	341(93.7)	23(6.3)	359(49.7)	5(45.5)	340(50.4)	24(41.4)
**Soft drink consumption**	No	578(85.5)	98(14.5)	573(84.4)	103(15.2)	614(90.8)	62(9.2)	635(93.9)	41(6.1)	665(92.1)	11(100.0)	623(92.3)	53(91.4)
	Yes	45(78.9)	12(21.1)	45(78.9)	12(21.1)	51(89.5)	6(10.5)	54(94.7)	3(5.3)	57(7.9)	0(0.0)	52(7.7)	5(8.6)
**Chocolate consumption**	No	510(85.0)	90(15.0)	505(84.2)	95(15.8)	544(90.7)	56(9.3)	561(93.5)	39(6.5)	591(81.9)	9(81.8)	546(80.9)	54(93.1)
	Yes	113(85.0)	20(15.0)	113(85.0)	20(15.0)	121(91.0)	12(9.0)	128(96.2)	5(3.8)	131(18.1)	2(18.2)	129(19.1)	4^*^(6.9)
**Coffee consumption**	No	514(82.5)	85(77.3)	502(83.8)	97(16.2)	544(90.8)	55(9.2)	564(94.2)	35(5.8)	589(81.6)	10(90.9)	548(81.2)	51(87.9)
	Yes	109(17.5)	25(22.7)	116(86.6)	18(13.4)	121(90.3)	13(9.7)	125(93.3)	9(6.7)	133(18.4)	1(9.1)	127(18.8)	7(12.1)
**Tea consumption**	No	116(18.6)	27(24.5)	125(87.4)	18(12.6)	131(91.6)	12(8.4)	134(93.7)	9(6.3)	139(19.3)	4(36.4)	128(19.0)	15(25.9)
	Yes	507(81.4)	83(75.5)	493(83.6)	97(16.4)	534(90.5)	56(9.5)	555(94.1)	35(5.9)	583(80.7)	7(63.6)	547(81.0)	43(74.1)
**Smoking**	No	520(85.8)	86(14.2)	514(84.8)	92(15.2)	548(82.4)	58(85.3)	568(93.7)	38(6.3)	600(83.1)	6(54.5)	558(82.7)	48(82.8)
	Yes	103(81.1)	24(18.9)	104(81.9)	23(18.1)	117(17.6)	10(14.7)	121(95.3)	6(4.7)	122(16.9)	5^*^(45.5)	117(17.3)	10(17.2)
**Exercise**	No	412(66.1)	67(60.9)	403(84.1)	76(15.9)	429(89.6)	50(10.4)	446(64.7)	33(75.0)	470(65.1)	9(81.8)	434(64.3)	45(77.6)
	Yes	211(33.9)	43(39.1)	215(84.6)	39(15.4)	236(92.9)	18(7.1)	243(35.3)	11(25.0)	252(34.9)	2(18.2)	241(35.7)	13^*^(22.4)

*: *p* < 0.05

**Table 4:** The association between different reproductive outcomes with mood status

Variables n (%)		Infertility		SAB		Stillbirth		preterm labor		IUGR		LBW	
		No	Yes	No	Yes	No	Yes	No	Yes	No	Yes	No	Yes
**Anxiety**	No	337(87.3)	49(12.7)	327(84.7)	59(15.3)	346(89.6)	40(10.4)	372(96.4)	14(3.6)	378(52.4)	8(72.7)	362(53.6)	24(41.4)
	Yes	286(82.4)	61 (17.6)	291(83.9)	56(16.1)	319(91.9)	28(8.1)	317(91.4)	30^*^(8.6)	344(47.6)	3(27.3)	313(46.4)	34(58.6)
**Nervous**	No	402(84.1)	76(15.9)	409(85.6)	69(14.4)	431(90.2)	47(9.8)	459(96.0)	19(4.0)	469(65.0)	9(81.8)	446(66.1)	32(55.2)
	Yes	221(86.7)	34(13.3)	209(82.0)	46(18.0)	234(91.8)	21(8.2)	230(90.2)	25^*^(9.8)	253(35.0)	2(18.2)	229(33.9)	26(44.8)
**Depression**	No	339(85.6)	57(14.4)	331(83.6)	65(16.4)	358(90.4)	38(9.6)	379(95.7)	17(4.3)	393(54.4)	3(27.3)	375(55.6)	21(36.2)
	Yes	284(84.3)	53(15.7)	287(85.2)	50(14.8)	307(91.1)	30(8.9)	310(92.0)	27^*^(8.0)	329(45.6)	8(72.7)	300(44.4)	37^*^(63.8)
**No moodDisturbance**	No	475(84.4)	88(15.6)	472(83.8)	91(16.2)	521(92.5)	42(7.5)	522(92.7)	41(7.3)	555(76.9)	8(72.7)	513(76.0)	50(86.2)
	Yes	148(87.1)	22(12.9)	146(85.9)	24(14.1)	144(84.7)	26^*^(15.3)	167(98.2)	3^*^(1.8)	167(23.1)	3(27.3)	162(24.0)	8(13.8)

*: *p* < 0.05

**Table 5:** The association between different reproductive outcomes with past medical history and reproductive history

Variables n (%)		Infertility		SAB		Stillbirth		preterm labor		IUGR		LBW	
		No	Yes	No	Yes	No	Yes	No	Yes	No	Yes	No	Yes
**Chronic disease**	No	544(84.2)	102(15.8)	571(88.4)	75(11.6)	599(92.7)	47(7.3)	619(95.8)	27(4.2)	638(88.4)	8(72.7)	608(90.1)	38(65.5)
	Yes	79(90.8)	8(9.2)	47(54.0)	40^*^(46.0)	66(75.9)	21^*^(24.1)	70(80.5)	17^*^(19.5)	84(11.6)	3(27.3)	67(9.9)	20^*^(34.5)
**Family marriage**	No	501(85.2)	87(14.8)	500(85.0)	88(15.0)	541(92.0)	47(8.0)	577(94.7)	31(5.3)	579(80.2)	9(81.8)	544(80.6)	44(75.9)
	Yes	122(84.1)	23(15.9)	118(81.4)	27(18.6)	124(85.5)	21^*^(14.5)	132(91.0)	13(9.0)	143(19.8)	2(18.2)	131(19.4)	14(24.1)
**Irregular menstruation**	No	361(87.2)	53(12.8)	363(87.7)	51(12.3)	377(91.1)	37(8.9)	390(94.2)	24(5.8)	407(56.4)	7(63.6)	375(55.6)	39(67.2)
	Yes	262(82.1)	57(17.9)	255(79.9)	64^*^(20.1)	288(90.3)	31(9.7)	299(93.7)	20(6.3)	315(43.6)	4(36.4)	300(44.4)	19(32.8)
**Gynecologic cause**	No	423(84.8)	76(15.2)	498(80.6)	1(0.9)	495(99.2)	4(0.8)	499(100.0)	0(0.0)	499(69.1)	0(0.0)	499(73.9)	0(0.0)
	Yes	200(85.5)	34(14.5)	120(19.4)	115^*^(99.1)	170(72.6)	64^*^(27.4)	190(81.2)	44^*^(18.8)	223(30.9)	11^*^(100.0)	176(26.1)	58^*^(100.0)
**OCP**	No	445(84.4)	80(15.2)	453(73.3)	72(62.6)	476(90.7)	49(9.3)	492(93.7)	33(6.3)	517(71.6)	8(72.7)	481(71.3)	44(75.9)
	Yes	178(85.6)	30(14.4)	165(26.7)	43^*^(37.4)	189(90.9)	19(9.1)	197(94.7)	11(5.3)	205(28.4)	3(27.3)	194(28.7)	14(24.1)
**PCO**	No	622(85.0)	110(15.0)	617(99.8)	115(100.0)	664(99.8)	68(100.0)	688(99.9)	44(100.0)	721(99.9)	11(100.0)	674(99.9)	58(100.0)
	Yes	1(100.0)	0(0.0)	1(0.2)	0(0.0)	1(0.2)	0(0.0)	1(0.1)	0(0.0)	1(0.1)	0(0.0)	1(0.1)	0(0.0)

*: *p* < 0

**Table 6:** Related factors of reproductive outcomes and breast feeding period

Stillbirth Adjusted OR (CI95%)		LBW Adjusted OR (CI95%)		Breast feeding period Adjusted OR (CI95%)		Infertility Adjusted OR (CI95%)		SAB Adjusted OR (CI95%)		preterm labor Adjusted OR (CI95%)	
**Income**	1.07 (1.02–1.11)	**Depression**	2.29 (1.29–4.08)	**Gynecologic cause**	6.88 (4.49–9.26)	**Job title**	2.6 (1.02–6.63)	**Working hour**	1.05 (1.01–1.08)	**Nervous**	2.87 (1.52–5.43)
**Solvents**	2.24 (1.07–4.7)	**Chronic disease**	3.95 (2.04–7.65)	**Working hour**	-0.14 (-0.26–0.03)	**Socioeconomic status**	0.46 (0.25–0.85)	**Irregular mens**	1.92 (1.14–3.24)	**Chronic** **disease**	6.1 (3.11–11.98)
**Mood disturbance**	3.28 (1.7–6.34)	**Gynecologic cause**	2.759 (1.36–5.6)	**Shift work**	-5.72 (-8.39–3.05)	**Fast food consumption**	1.8 (1.06–3.05)	**Gynecologic cause**	8.4 (1.14–3.24)	**Constant**	0.006
**Gynecologic cause**	2.84 (1.47–5.49)	**Caffeinated drinks**	2.15 (1.04–4.44)	**Depression**	-2.59 (-4.82–0.35)	**Constant**	0.08	**Constant**	0.00		
**Family marriage**	2.24 (1.15–4.36)	**Constant**	0.031	**Constant**	24.3						
**Constant**	0.005										

## Discussion

This study aimed to provide the association between different occupational hazards in the healthcare industry and reproductive health, pregnancy outcomes, and lactation period. Healthcare workers are exposed to various occupational hazards such as anesthetic gas, solvents, antineoplastic drugs, ionizing and non-ionizing radiation, ergonomic, shift work, stress, violence, and biological hazards, which can cause some reproductive complications such as infertility, spontaneous abortion (SAB), stillbirth, preterm labor, and low birth weight.

Globally, infertility is a prevalent issue that affects over 186,000,000 couples in the world, and most of its social effect is on women [12, 13]. The incidence rate of infertility in Canada was 11.5–15.7% [14]. The infertility rate has been reported at 12.5%, 15.5%, and 25% in Britain, the U.S., and China, respectively [15]. The prevalence of infertility in Iran was 7.88%, according to a systematic review and meta-analysis survey conducted by Marzieh Saei (2020) et al. [16]. In our study, the frequency of infertility was 15%. The associated factors were low socioeconomic status, working as clinical staff, and consumption of fast-food (unsafe diet).

Similar to our results, Sarah L. Berga, M.D. (2016), concluded that couples with a higher annual income had higher conception rates, [17] It means that low socioeconomic status may increase infertility rates. Thomas H. Connor (2014) et al. pointed out in his study that healthcare workers can be exposed to chronic occupational exposures like antineoplastic drugs and appear to have an increased risk of adverse reproductive outcomes because of the gonadal toxicity of the drugs (injury to ovarian follicles which result in ovarian volume reduction and fibrosis which cause amenorrhea.), especially with exposures during the first trimester of pregnancy [18, 19]. Sarac M (2017) presented that consumption of fried foods is a predisposing factor for infertility [20]; the same results about the association between infertility and fast food were found in our study. This observation aligned with another research that mentioned that a high saturated fat diet is associated with infertility [21]. It is known that higher body mass index (BMI) and obesity [21, 22], higher age of marriage [20], sexually transmitted disease [23, 24], and coping with stress affect the average trend of reproduction. Furthermore, exposure to tobacco smoke, alcohol consumption, and air pollution are associated with early reproductive outcomes such as fertilization. This point is noteworthy because we could not find all known risk factors for infertility due to the low frequency of different hazards in our study.

Spontaneous abortion (SAB) or miscarriage is one of the most common pregnancy complications [25]. It is a medical problem that may adversely influence the emotional aspects of couples that wish to have a child. The incidence of spontaneous abortion is reported to be between 10 and 20% [9]. In India (2015), the prevalence of spontaneous abortion was 7.2% [26]. In the United States, 15% of known pregnancies end with spontaneous abortion [27]. In Iran, different rates of spontaneous abortion (7–25%) have been reported, 7.46%, 8.3%, 9%, and 25.7% in sequence in Shiraz [28], Ardabil [29], Tehran [30], and Kermanshah [31]. This diversity may be explained by women’s income and educational level variations. The frequency of SAB was 15.7% in our study. The associated variables of spontaneous abortion in our study were higher working hours, increasing age, irregular menstruation, and gynecologic disorders. The most reason for spontaneous abortion (more than half of the cases) are genetic disorders and chromosomal abnormalities, but some other factors which are related to SAB are age > 35, consumption of smoking and alcohol, physical stress, and exposure to antineoplastic drugs and heavy metals [32, 33]. Physical strain around implantation was associated with spontaneous abortion [34]. Elizabeth A et al. concluded that women working more than 40 h/week during the first trimester are at increased risk of spontaneous abortion compared with women working less than 40 h [35]. Although the previous studies smoking cigarettes and alcohol consumption were independent risk factors in our study, that could be due to underreporting.

Preterm labor is a fundamental public health problem leading to neonatal morbidity and mortality [36]. The prevalence of preterm birth was 10.9% and 12% in Australia and the United States, respectively [37]. According to a systematic review and meta-analysis in 2015 in Iran, the prevalence of preterm birth was 9.2% [38]. Compared to other studies, the prevalence of preterm delivery was lower in our study (6%). It could be due to higher access of hospital staff to public health resources and level of education in health care workers and good-income levels in contrast with the general population, which is directly affected by preterm delivery. History of previous preterm labor or family history of prematurity, low maternal body mass index (BMI), low general health status, black race, history of disease during pregnancy, decreased amniotic fluid, multiple pregnancies, and infertility are some of the mentioned preterm labor etiologies [39]. Our study’s related risk factors of preterm labor were chronic disease and nervousness. EK Çam et al. (2013) reported that chronic disease causes a higher rate of preterm labor [40]. In our study, maternal education was correlated to preterm labor, which was in line with the previous observations. According to our results, mothers with a lower educational degree had a higher risk of delivering a preterm baby. Former research mostly concluded that anxiety and depression were associated with preterm labor. A synergic action of psychological and biomedical factors on the secretion of placental corticotrophin-releasing factor is hypothesized [41].

The prevalence of stillbirth (infant death ≥ 22 weeks’ gestation) in different countries varies significantly, but according to a study in 2016, the stillbirth rate (SBR) is estimated to be 18.4 per 1000 births worldwide [42]. The average stillbirth rate was 7.42 per 1000 total births during 2014–2016 in Iran [43]. Although the statistics in Iran are lower than the world’s rates, variation may be due to socioeconomic inequalities and a lack of an appropriate healthcare registry system.

The prevalence of stillbirth in our study was 9.3%. The associated factors were higher income, family marriage, solvent exposure, and gynecologic disorders. Identifiable causes can be attributed to maternal, fetal, and placental conditions. The most critical related factors of stillbirth were preterm birth and post-term birth [26]. Of 96 studies in a systematic review (2011), Maternal high BMI (body-mass index > 25 kg/m2) was the highest-ranking modifiable risk factor of stillbirths. Maternal age (> 35 years) and maternal smoking increased the stillbirth risk. Another critical factor was a chronic disease of the mother (diabetes and hypertension). Placental abruption is a known cause of stillbirth [44]. parents who lived in the most deprived locations encountered stillbirth more [45, 46]. which also supports another study, reported that 98% of stillbirths occurred in low-income and middle-income countries, similar to our research. Both null parity and multiparity (> 3) were the risk factors of this issue, which has a U shape diagram [9] 98% of stillbirths occurred in low-income and middle-income countries [47].

Low birth weight is a significant public health problem and a predictor of infant mortality. According to UNICEF statistics, the global rate of LBW stands at 17% (6% in industrialized countries vs. 21% in developing ones) [48]. The prevalence of LBW was reported at 8.8% and 9.4% in Yazd and the south of Iran, respectively [42]. Consanguineous marriage, pregnancy age < 18 and > 35 years old, maternal medical risk factors, the female sex of the fetus, and lower maternal education level are known as LBW risk factors [49]. There are social disadvantages such as low socioeconomic status, low education, poor nutrition, and low body mass index responsible for these results in younger mothers; however, in older mothers, biological factors such as chromosomal anomalies, preeclampsia, and diabetes are responsible for this issue [50]. In addition, the incidence of LBW in low-income societies was reported more than twice compared to middle-income countries [51]. We found that depression, gynecologic disorders, chronic disease, and consumption of caffeinated drinks as correlated risk factors. Similar to our results, Golestan M et al. (2011) mentioned that maternal diseases, especially hypertension, could increase LBW rates [48, 52]. Similar to other studies, it was shown in logistic regression that depression has an association with LBW [53, 54].

Breastfeeding is one of the infants’ health indexes. WHO mentioned that children who are breastfed are healthier and also are better in their education. Breast milk gives infants the best start in life and provides immunological protection and critical nutrients for brain development [55]. The breastfeeding period was significantly decreased in mothers who have shift work. About 5.3% of our participants, with at least one child, did not breastfeed, and 18.2% breastfed their children less than six months. According to our data, the mothers who were shift workers breastfeed their children for about 13 months, whereas this rate is about 20 months in office workers (5.7 months more than shift workers). Also, Breastfeeding length decreased in staff with prolonged working hours and depression.

## Conclusion

The study revealed that chemical and ergonomic exposures have associations with stillbirth and spontaneous abortion, respectively. We found no considerable increase in the risk of reproductive outcomes with working in shifts. However, the breastfeeding period was significantly decreased in shift worker mothers. Psychiatric disorders were associated with preterm labor, low birth weight, and stillbirth in sequence with nervousness, depression, and mood disturbance. Moreover, depression was found to be related to breastfeeding length. Furthermore, socioeconomic status is influenced by stillbirth and infertility. In addition, we found a link between job titles and infertility.

### Strengths and limitations of the study

In our study, we benefited from a large sample size. Another positive point of the current study was an investigation of multiple exposures and outcomes.

The cross-sectional nature of this study limits its generalizability and causal relationship. Also, the exposure assessment was based on self-reports, so we lack information about exposure measurement and the implementation of engineering control to reduce exposure. Another influence of self-report data gathering is the possibility of biased recall. And due to lack of psychological measurements we couldn’t evaluate the psychological distress and stress affecting the participants. Moreover, participants may not be aware of all their exposures, and asking one about the exposure may not provide sufficiently accurate information.

### Recommendations

Three variables could be considered as the potentially most modifiable factors. However, they have different implications for prevention. One of the modifiable risk factors is mood disturbance, which is related to some reproductive and pregnancy outcomes. Holding emotion and stress management courses can improve the psychiatric status of hospital staff.

The second one is chemical, physical, and ergonomic hazards. The workplace should be safe for all workers. Thus, preventing occupational hazards must be a primary goal for healthcare workers, and assessment of workplace harms is necessary.

Exposure reduction/ elimination, which is the most desirable, substitution with safer materials and improved engineering controls are suggested for decreasing/ preventing encounters with chemical exposures like solvents, antineoplastic drugs, and anesthetic gases, and even physical hazards such as ionizing and non-ionizing radiations. Moreover, staff should be trained in self-safety protection. Finally, PPE (personal protective equipment) should be in access, and more importantly, temporary job transfer could be helpful in situations where a reproductive hazard exists. However, the problem may occur when there is no non-exposed job location, especially in some workplaces such as hospitals; thus, paid leave should be considered when there is a high-risk situation, and exposure management is not possible. To manage ergonomic hazards such as physical loads and prolonged working hours, hospital heads are recommended to modify working hours in women of reproductive age, especially in the first trimester of pregnancy, to avoid reproductive complications. On the other hand, the risk of spontaneous abortion due to high physical demand can be reduced by handling working hours and paid leave.

The last one is shift work that should be modified for mothers with newborn babies and considering hourly paid leave for them to breastfeed their children.

## Data Availability

Data and material will be available upon email to the corresponding author.

## Abbreviations

BLS: Bureau of Labor Statistics
BMI: Body mass index
CDC: Centers for Disease Control and Prevention
IUGR: Intrauterine growth retard
LBW: Low birth weight
SAB: Spontaneous abortion
SD: Standard deviation
WHO: World Health Organization
